# Combined Supplementation of *Clostridium butyricum* and *Bifidobacterium infantis* Diminishes Chronic Unpredictable Mild Stress-Induced Intestinal Alterations via Activation of Nrf-2 Signaling Pathway in Rats

**DOI:** 10.3390/ijms24098264

**Published:** 2023-05-05

**Authors:** Sabiha Fatima, Haifa Altwaijry, Mahmoud M. A. Abulmeaty, Manal Abudawood, Nikhat J. Siddiqi, Reem Hamoud Alrashoudi, Sarah Alsobaie

**Affiliations:** 1 Department of Clinical Laboratory Sciences, College of Applied Medical Sciences, King Saud University, Riyadh 11433, Saudi Arabia; haaltwaijry@ksu.edu.sa (H.A.); ralrashoudi@ksu.edu.sa (R.H.A.); salsobaie@ksu.edu.sa (S.A.); 2 Department of Community Health Sciences, College of Applied Medical Sciences, King Saud University, Riyadh 11362, Saudi Arabia; mabulmeaty@ksu.edu.sa; 3 Chair of Medical and Molecular Genetics Research, Department of Clinical Laboratory Sciences, College of Applied Medical Sciences, King Saud University, Riyadh 11433, Saudi Arabia; 4 Department of Biochemistry, College of Science, King Saud University, Riyadh 11495, Saudi Arabia; nikhat@ksu.edu.sa

**Keywords:** *Clostridium butyricum*, *Bifidobacterium infantis*, CUMS, intestine, Nrf-2 signaling

## Abstract

Exposure to long-term chronic unpredictable mild stress (CUMS) can cause redox imbalance and inflammation, which may affect the integrity of the gut barrier. The present study was conducted to investigate the effects of a probiotics bacterium mixture, including *Clostridium butyricum (C. butyricum)* and *Bifidobacterium infantis (B. infantis)*, on the intestinal homeostasis in rats exposed to multiple low-intensity stressors for 28 days. The mechanism of CUMS-induced altered intestinal homeostasis was evaluated by focusing on the nuclear factor-E2-related factor-2 (Nrf-2) pathway. In contrast to the CUMS group, probiotic mixture supplementation significantly (*p* < 0.01) reversed the stress-induced elevated corticosterone level, protein and lipid oxidation, and increased enzymatic and non-enzymatic antioxidant levels, as well as upregulated Nrf-2/HO-1 pathway. Probiotics supplementation further significantly (*p* < 0.01) decreased the CUMS-induced inflammation, altered T-lymphocyte levels, and suppressed the protein expression of nuclear factor kappa B (NF-κB) in rat intestines. Improvement in histological changes and intestinal barrier integrity further validate the beneficial effects of probiotic mixtures on CUMS-induced altered intestinal morphology. In conclusion, our results suggest that the combination of *C. butyricum* and *B. infantis* significantly attenuated CUMS-induced oxidative stress, inflammation, and T-lymphocyte modulation by upregulating Nrf-2/HO-1 signaling and inhibiting NF-κB expression in rat intestine.

## 1. Introduction

Stress-related intestinal disorder is becoming common in modern human life. In both humans and animals, stress is a major risk factor for gastrointestinal diseases [1,2]. The effect of chronic unpredictable mild (CUMS) stress has been studied in many animal models, as they respond to stress in similar ways to humans [3,4]. The impact of stress on the clinical course of some intestinal inflammatory diseases, such as irritable bowel syndrome and inflammatory bowel disease, is highly prevalent in developed countries [1]. Some clinical studies suggest that minor daily stressors can adversely affect gastrointestinal health [5,6].

Tight junction proteins, such as claudins, zonula occludens, and occludin, are essential to maintaining epithelial barrier integrity [7]. Several in vivo studies have shown that CUMS destroys tight junction proteins, increasing intestinal permeability by damaging the mucous layer [3,8]. This allows harmful bacteria and endotoxins to enter intestinal cells and the bloodstream, causing inflammation and tissue damage, and increasing the risk of gastrointestinal infections and inflammation-related diseases [1,9]. On exposure to multiple unpredictable stressors, dysregulation of the gut–brain axis stimulates the release of the stress hormone corticosterone, which, by altering the structure and function of the mucosal barrier, induces intestinal disorder [10,11]. In addition to causing gastrointestinal disorders, chronic inflammation associated with long-term stress has also been linked to diabetes, autoimmune diseases, cardiovascular disease, and various cancers [12,13]

Studies show that chronic stress induces oxidative stress, inflammation, and immune dysregulation by altering gut flora [14,15]. During stress, cells are prone to oxidative damage due to the excessive generation of reactive oxygen species (ROS) that lead to cell death and tissue dysfunction [16]. An imbalanced oxidative status is associated with enhanced inflammatory responses [17]. In addition to promoting pro-inflammatory responses, chronic stress substantially affects immune function, which eventually increases the risk of infection. Numerous studies have reported that stress can alter leukocyte numbers and distribution in the body, which can suppress protective immune responses [18]. The attenuation of oxidative stress and inflammation is crucial to maintain the immune–metabolic balance of the body [18]. The transcription factor Nrf-2, a cellular oxidative stress response regulator, maintains redox homeostasis and protects the cells by activating antioxidant gene expression [19]. By activating antioxidant gene expression and blocking pro-inflammatory cytokines, Nrf-2 has been reported to reduce oxidative stress and inflammation, which can ensure healthy gastrointestinal integrity [20].

As reported earlier, disruption of intestinal bacterial microbiota during stress plays a crucial role in gut pathophysiology. Probiotics contain live nonpathogenic bacteria which colonize the gastrointestinal tract. These bacteria reduce oxidative stress, inflammation, and other stress-induced changes in the gut microenvironment [21,22]. Maintenance of healthy gut flora by probiotics, through immunity regulation, can exert beneficial therapeutic effects on gastrointestinal infections and inflammation-related diseases [23]. *Clostridium butyricum* (*C. butyricum*), a Gram-positive anaerobic spore-forming bacteria commonly found in the human and animal gut, is used as a probiotic to treat and prevent gastrointestinal disorders [24]. According to studies, certain strains of *C. butyricum* have antioxidant and anti-apoptotic properties and can boost the immune system [25,26,27]. Among probiotics, a Gram-positive dominant colonic microbiota *Bifidobacterium infantis* (*B. infantis*) makes up almost 80% of infant and 25% of adult fecal bacterial flora [28]. Some strains of *Bifidobacterium* have potent anti-inflammatory and immune-modulating effects [29]. The effectiveness of *B. infantis* as a probiotic agent has been studied across a wide spectrum of gastrointestinal disorders [30,31]. However, the mechanism associated with the probiotics, including the mixture of *C. butyricum* and *B. infantis*, in preventing CUMS-associated intestinal disorder remains unclear.

Therefore, a CUMS model in the current study was used to examine the combined effect of probiotic bacteria *C. butyricum* and *B. infantis* on stress-induced altered gut homeostasis. The study investigates the effect of *C. butyricum* and *B. infantis* on stress-induced altered glucocorticoid levels, intestinal oxidative stress, inflammation, immune markers, and gut permeability. Additionally, the study assessed the effect of *C. butyricum* and *B. infantis* on the molecular mechanism and signaling pathways that regulate oxidative stress and inflammation.

## 2. Results

### 2.1. Effect of CUMS on Body Weight Change

**Figure 1:**
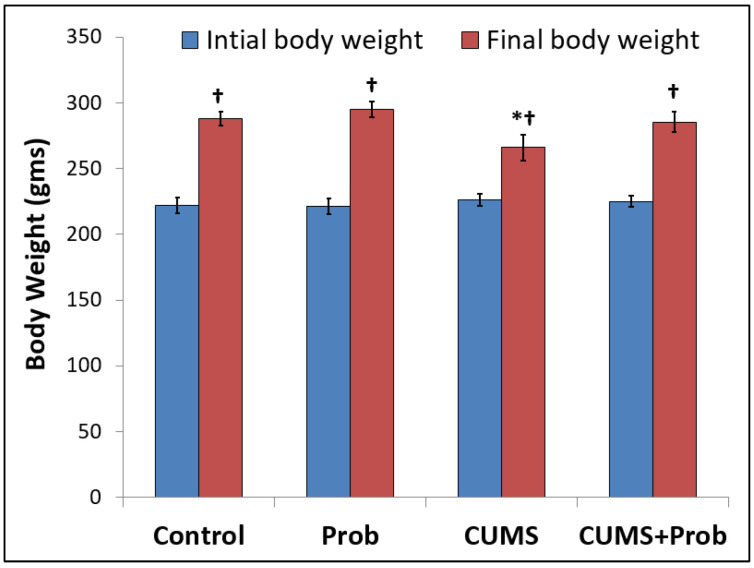
Change in the body weight of rats exposed to chronic unpredictable mild stress (CUMS) for 28 days with or without daily supplementation with a probiotic (Prob) mixture. Results are mean ± SEM of 6 rats per group. † indicates a significant difference (*p* < 0.05) compared to initial body weight, * indicates a significant difference (*p* < 0.05) of CUMS from the control and probiotic (Prob) group; one-way ANOVA followed by Tukey’s post hoc test.

During the experimental period, the CUMS-exposed rats with or without probiotics supplementation were weighed, and their initial and final body weights were compared to those of the control group. The control group and non-stressed group with probiotics supplementation were physically active and showed normal weight gain. In contrast to the control group, the CUMS-exposed rats showed longer sleep duration and slower activity during the experimental period. The CUMS-exposed rats showed small but significantly lower weight gain relative to the control and probiotics-supplemented group (Figure 1). However, rats supplemented with a probiotic mixture during exposure to CUMS showed improvement in weight gain.

### 2.2. Effect of Probiotics on CUMS-Induced Altered Plasma Corticosterone Level

**Figure 2:**
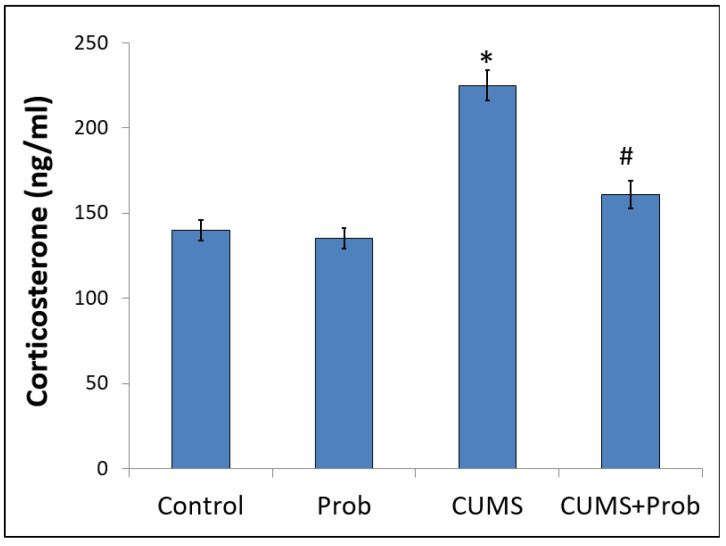
Daily supplementation with the probiotic mixture improved plasma corticosterone levels in rats exposed to chronic unpredictable mild stress (CUMS) for 28 days. The values are mean ± S.E.M (n = 6). * indicates a significant difference (*p* < 0.001) of CUMS from the control and probiotic (Prob) group, # indicates a significant difference (*p* < 0.01) of CUMS+Prob from CUMS group; one-way ANOVA followed by Tukey’s post hoc test.

Corticosterone, a major hormone released in rodents in response to stress, was measured in the plasma of rats with or without probiotics supplementation. As shown in Figure 2, exposure to CUMS significantly increased (*p* < 0.001) plasma corticosterone levels compared to the control group, while daily supplementation of the probiotic mixture significantly (*p* < 0.01) reduced the plasma corticosterone levels. An insignificant change in plasma corticosterone levels was observed between the non-stressed probiotics-treated group and the control group.

### 2.3. Effect of Probiotics on Stress-Induced Altered Inflammatory Markers

**Figure 3:**
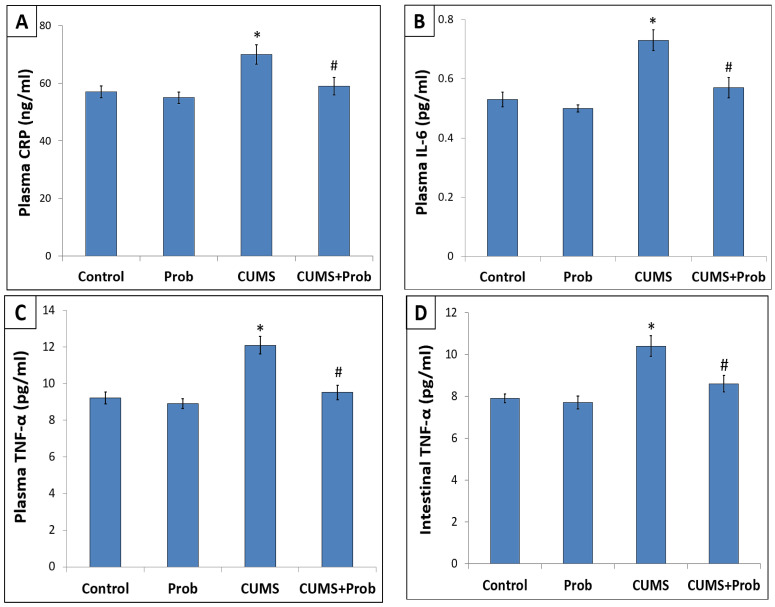

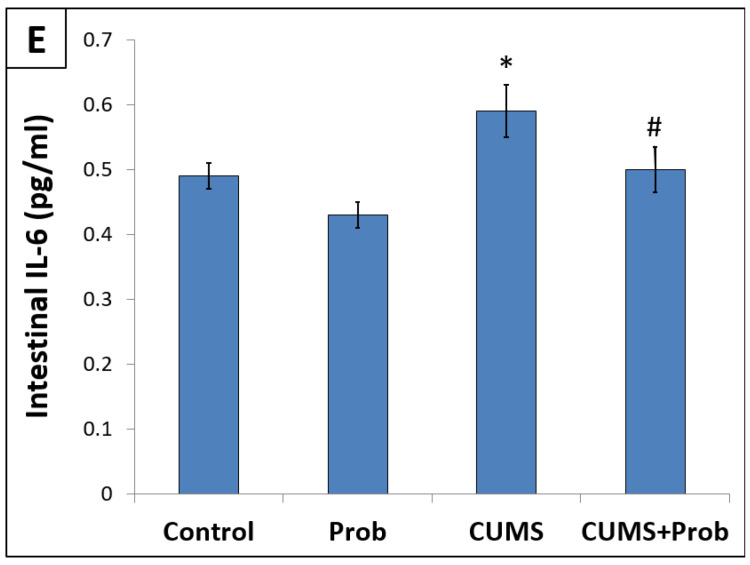
Supplementation with a probiotic (Prob) mixture reduced the inflammatory markers in the plasma (**A**–**C**) and intestine (**D**,**E**) of rats exposed for 28 days to chronic unpredictable mild stress (CUMS). The values are mean ± S.E.M (n = 6). * indicates a significant difference (*p* < 0.01) of CUMS from the control and probiotic (Prob) group, # indicates a significant difference (*p* < 0.05) of CUMS+Prob from CUMS group; one-way ANOVA followed by Tukey’s post hoc test.

Figure 3 shows the levels of inflammatory markers in control and CUMS-exposed rats, with or without supplementation with a probiotic mixture. Plasma CRP, an acute-phase protein, is commonly used as a potential indicator of systemic inflammation. The stressed rats showed a significant (*p* < 0.01) increase in plasma CRP level compared with the control group (Figure 3A). Moreover, exposure to CUMS showed significantly increased (*p* < 0.01) levels of the inflammatory cytokines TNF-α and IL-6 in plasma (Figure 3B,C) and intestinal tissue homogenates compared to the control group (Figure 3D,E). However, compared with the CUMS-exposed group, rats receiving the probiotic mixture daily during CUMS exposure showed significant attenuation (*p* < 0.05) of inflammatory markers in plasma and intestine.

**Figure 4:**
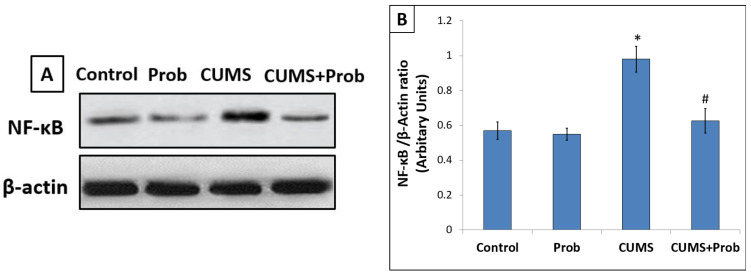
Effect of probiotics supplementation on the protein expression of NF-κB in the intestine of rats exposed to 28 days of chronic unpredictable mild stress (CUMS). (**A**): Blot bands of protein expression of NF-κB in the intestine; (**B**): The protein density quantified relative to β-actin used as an internal control. The values are mean ± S.E.M (n = 6). * indicates a significant difference (*p* < 0.001) of CUMS from the control and probiotics (Prob) group, # indicates a significant difference (*p* < 0.01) of CUMS+Prob compared to CUMS group; one-way ANOVA followed by Tukey’s post hoc test.

Along with the inflammatory cytokines, the protein expression of NF-κB, a transcription factor that induces the gene expression of many inflammatory cytokines, was estimated in each group. In CUMS-exposed group, the protein expression of NF-κB was significantly higher (*p* < 0.001) compared to the control group. In contrast to the stress-exposed rats, the CUMS-exposed group supplemented with a daily probiotic mixture showed significantly (*p* < 0.01) reduced levels of NF-κB protein expression (Figure 4).

Unstressed rats supplemented with a probiotic mixture showed insignificant reduced levels of inflammatory markers and NF-κB protein expression compared to the control group.

### 2.4. Effects of Probiotics on CUMS-Induced Altered Intestinal Redox Status

**Figure 5:**
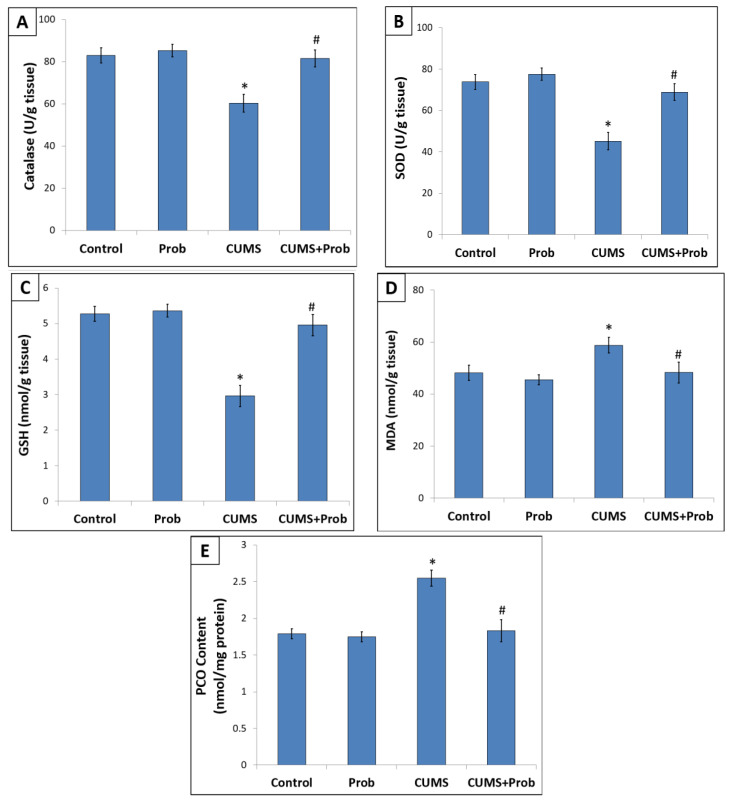
The effect of probiotics supplementation on (**A**): Catalase activity, (**B**): Superoxide dismutase (SOD) activity, (**C**): Reduced glutathione content, (**D**): Malondialdehyde (MDA) level, and (**E**): Protein carbonyl (PCO) content in the intestine of the rats exposed to CUMS for 28 days. The values are mean ± S.E.M (n = 6). * indicates a significant difference (*p* < 0.01) of CUMS from the control and probiotic (Prob) group, # indicates a significant difference (*p* < 0.01) of CUMS+Prob from CUMS group; one-way ANOVA followed by Tukey’s post hoc test.

Figure 5 reveals oxidative stress markers in the intestine of rats exposed to 28 days of CUMS with or without probiotics supplementation. In the intestinal mucosa, CUMS exposure induced a significant decrease in the non-enzymatic antioxidant GSH.

Contrary to the control group, exposure to CUMS induced a significant decrease in the non-enzymatic antioxidant GSH (Figure 5C), along with a marked increase in lipid and protein peroxidation products MDA (Figure 5D) and PCO content (Figure 5E) in the intestinal mucosa. Moreover, CUMS suppressed the activities of intestinal antioxidant enzymes like catalase and SOD as compared to the control group (Figure 5A,B).

However, daily supplementation of the probiotics to stress-exposed rats restored the altered levels and/or activities of enzymatic and non-enzymatic antioxidants, and reduced the levels of MDA and PCO in the intestinal mucosa compared to rats exposed to CUMS without supplements.

In comparison to the control group, daily administration of the probiotics showed a better antioxidant level in the non-stressed rats.

### 2.5. Effect of Probiotics on the T-Lymphocytes in the Intestinal Mucosa of the Rats Exposed to Chronic Unpredictable Stress

**Figure 6:**
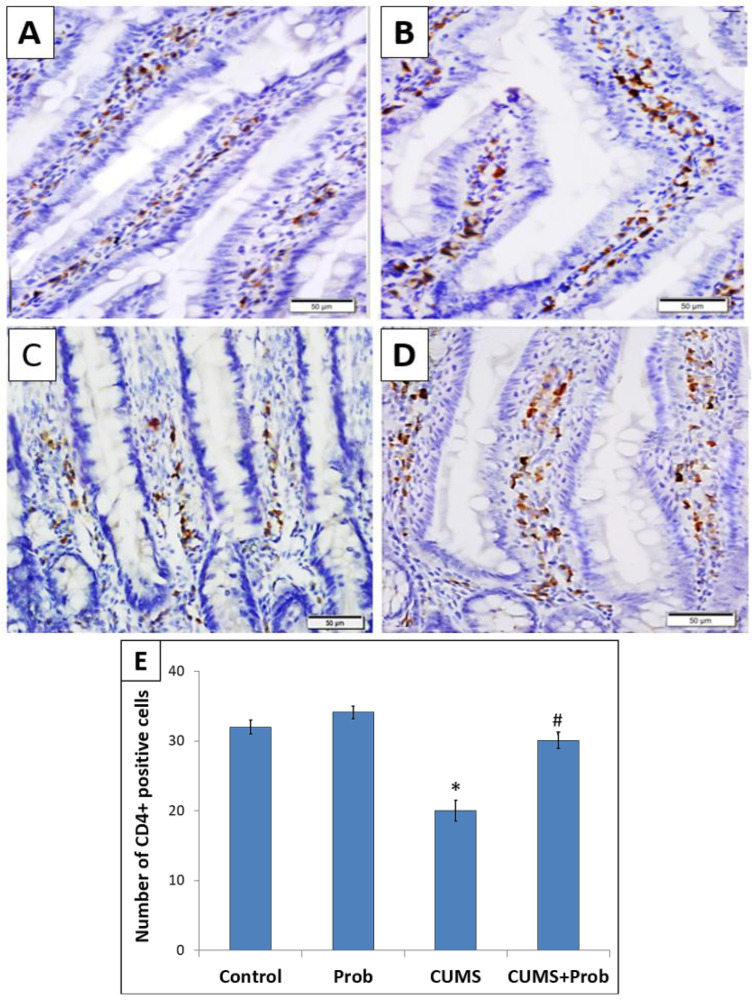
The effect of probiotics supplementation on the expression of CD-4+ T-lymphocytes in the intestine of (**A**): control, (**B**): probiotics (Prob)-supplemented group; (**C**): chronic unpredictable mild stress (CUMS)-exposed group, and (**D**): CUMS+Prob group. Scale bar 50, magnification 400×. (**E**). Bar graph showing the relative number of CD-4+ positive cells in the intestine of rats exposed to CUMS with or without probiotics supplementation. Each CD4+T-lymphocyte stained section by the positive cells was counted manually in 20 random fields at a magnification of 400×. * indicates a significant difference (*p* < 0.001) of CUMS from the control and probiotics (Prob) group, # indicates a significant difference (*p* < 0.001) of CUMS+Prob from CUMS group; one-way ANOVA followed by Tukey’s post hoc test.

**Figure 7:**
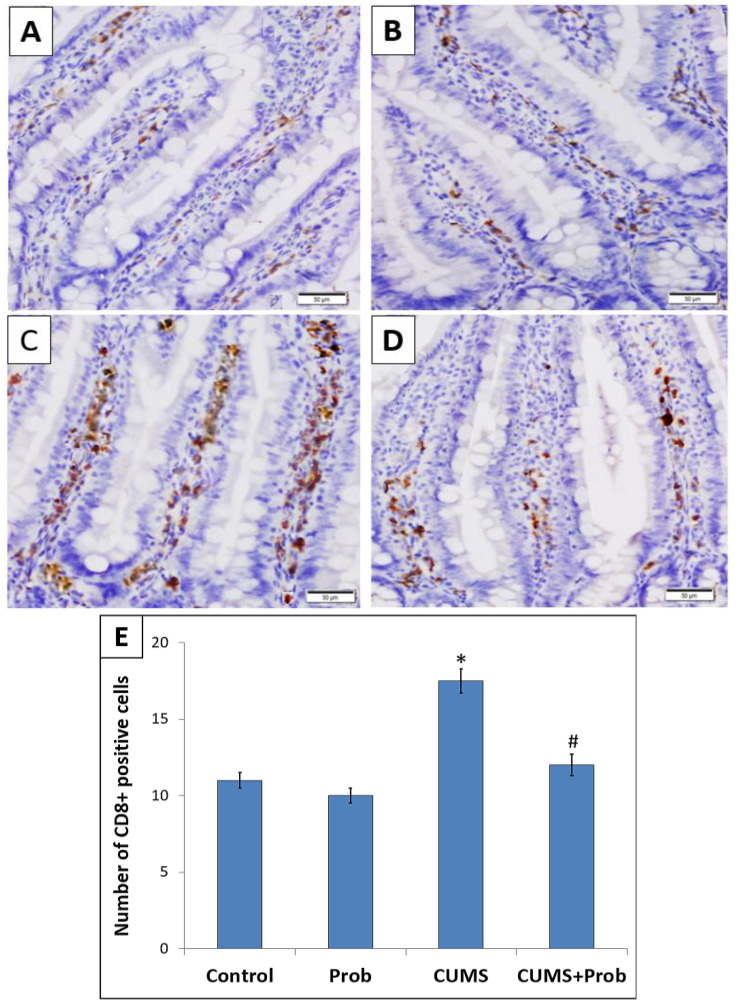
The effect of probiotics supplementation on the expression of CD-8+ T -lymphocytes in the intestine of (**A**): control, (**B**): probiotics (Prob)-supplemented group; (**C**): chronic unpredictable mild stress (CUMS) exposed group, and (**D**): CUMS+Prob group. Scale bar 50 µm, magnification 400×. (**E**). Bar graph is showing the relative number of CD-8+ T-positive cells in the intestine of rats exposed to CUMS with or without probiotics supplementation. Each CD4+T-lymphocyte stained section by the positive cells was counted manually in 20 random fields at a magnification of 400×. * indicates a significant difference (*p* < 0.001) of CUMS from the control and probiotics (Prob) group, # indicates a significant difference (*p* < 0.01) of CUMS+Prob from CUMS group; one-way ANOVA followed by Tukey’s post hoc test.

Immunohistochemistry was performed to investigate the potential role of probiotics supplementation on CD4+ T-helper and CD8+ T-cytotoxic lymphocytes in the intestinal mucosa of the rats exposed to CUMS (Figure 6 and Figure 7). Exposure to CUMS significantly decreased (*p* < 0.001) the number of CD-4+ T-positive cells within the intestinal mucosa as compared to the control group (Figure 6). The CUMS-induced decrease of CD-4+ T-lymphocyte expression was associated with the increase (*p* < 0.001) in CD-8+ T-positive cells compared to the non-stressed control rats (Figure 7). However, probiotics supplementation daily for 28 days significantly (*p* < 0.01) restored the altered CD-4+ T- and CD-8+ T-lymphocyte levels in the intestinal mucosa of CUMS exposed rats. This indicates the potential role of probiotics in restoring the immune markers in rats exposed to CUMS. Probiotics administration daily to the non-stressed rats showed increased CD-4+ T- and CD- 8+ T-cells expression levels; however, the increase was not statistically significant compared to the control group.

### 2.6. The Effects of Probiotics Supplementation on the Gut Permeability of CUMS-Exposed Rats

**Figure 8:**
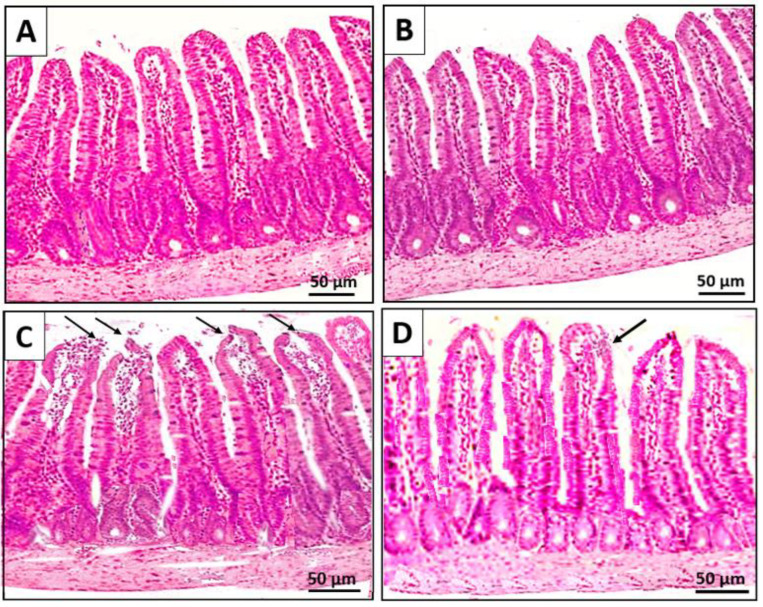
The effect of probiotics supplementation on intestinal histology in chronic unpredictable mild stress (CUMS) treated rats. The intestinal villi in (**A**): control and (**B**): probiotics supplemented groups look intact and tidier. (**C**): The intestine of the rats exposed to CUMS shows loose and denuded villi with damaged surface epithelium. The black arrow indicates a damaged mucosal layer of the villi. (**D**): Supplementation of probiotics during exposure to CUMS markedly improved intestinal histology with relatively intact structure, with occasional shedding of surface epithelium compared to the stress group. Scale bar 50 μm, magnification 200×.

To assess the effect of stress, an analysis of the gut structural integrity was performed. The intestinal mucosa of control rats showed intact villi with no pathological changes (Figure 8A). However, compared to controls, probiotics supplementation in the non-stressed group showed much healthier intestinal mucosa with intact villi covered with an epithelial layer (Figure 8B). The CUMS exposure resulted in the loss of epithelial surface with loose and denuded villi (Figure 8C). Supplementation of probiotics during the stress treatment significantly preserved the structure of intestinal villi, with improved intestinal histology showing closely arranged villi with intact surface epithelium (Figure 8D).

**Figure 9:**
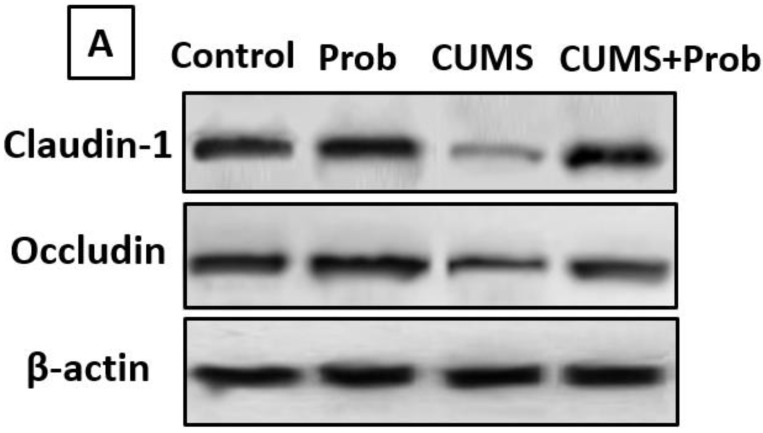

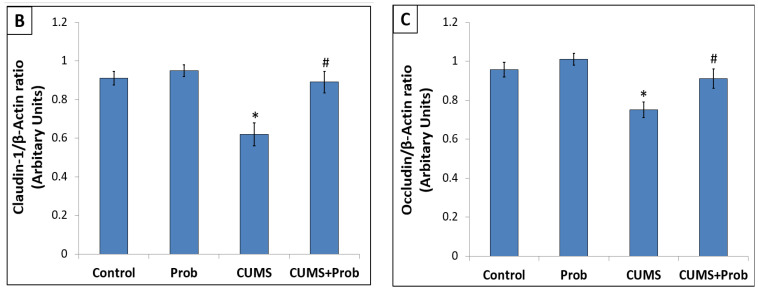
The relative protein expression of tight-junction proteins in intestinal tissue of rats that were exposed to CUMS with or without probiotics supplementation. (**A**): Blot bands of protein expression of claudin-1 and occludin in the intestine. The protein density of (**B**): claudin-1 and (**C**): occludin was quantified relative to β-actin, and used as an internal control. The values are mean ± S.E.M (n = 6). * indicates a significant difference (*p* < 0.01) of CUMS from the control and probiotic (Prob) group, # indicates a significant difference (*p* < 0.01) of CUMS+Prob from CUMS group; one-way ANOVA followed by Tukey’s post hoc test.

An important determinant of intestinal function is barrier integrity, which is determined by tight junction proteins. When compared to the control group, rats exposed to CUMS had decreased expression levels of occludin and claudin-1 proteins in the intestinal mucosa. However, compared to CUMS-exposed rats, the CUMS+Prob group showed increased occludin and claudin-1 protein expression levels (Figure 9).

### 2.7. Effect of Probiotics on the Nrf-2 Signaling Pathway in Rats Exposed to Chronic Unpredictable Stress

**Figure 10:**
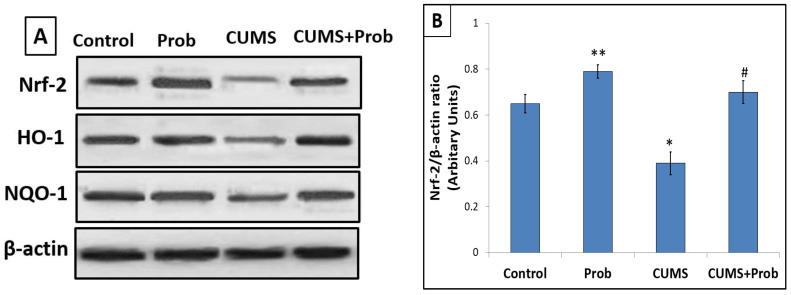

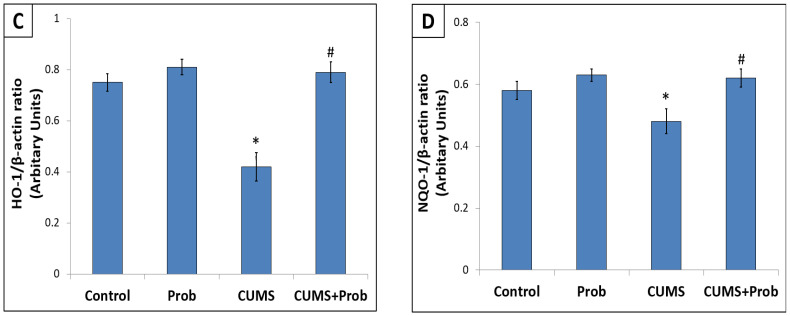
Effects of probiotics supplementation on the Nrf-2 signaling pathway in the intestine of the rats exposed to chronic unpredictable stress. (**A**): Blot bands of protein expression of Nfr-2, HO-1, and NQO-1 in the intestine. The protein density of (**B**): Nfr-2, (**C**): HO-1, (**D**): NQO-1 was quantified relative to β-actin, and used as an internal control. The values are mean ± S.E.M (n = 6). ** indicates a significant difference (*p* < 0.05) of Prob from the control group, * indicates a significant difference (*p* < 0.01) of CUMS from the control and probiotics (Prob) group, # indicates a significant difference (*p* < 0.01) of CUMS+Prob from CUMS group; one-way ANOVA followed by Tukey’s post hoc test.

Figure 10 shows the expression of Nrf-2 signaling pathway-related proteins in the control group and the rats exposed to CUMS with or without probiotic supplementation. Compared with the control group, exposure to stress significantly (*p* < 0.01) decreased the protein expression level of Nrf-2, HO-1, and NQO-1 in the intestine. Probiotics supplementation, however, significantly reversed the stress-induced reduction of Nrf-2, HO-1, and NQO-1 protein expression, while the non-stressed probiotics supplemented group showed significantly (*p* < 0.01) increased Nrf-2 and NQO-1 protein levels in comparison to the control group.

## 3. Discussion

Chronic unpredictable mild stress (CUMS) is the most clinically-relevant and frequently-used method for generating chronic stress in animal models, which is considered a more naturalistic representation of human physical and psychological stress aspects [32]. Chronic low levels of unpredictable stressful stimulation over a long period form the basis of the CUMS model. In the present study, CUMS treatment did not substantially alter the water and food intake in rats. However, after 28 days of the experimental period, rats exposed to CUMS showed significantly reduced weight gain compared to the control group (Figure 1). Our result was consistent with previous studies which reported that exposure to multiple chronic stressors may cause reduced weight gain in rodents because of loss of fat mass [33,34].

In non-stressed rats, corticosterone, the main glucocorticoid in rodents, appeared to have a basal circulatory level. However, the response to CUMS-induced aversive environment, characterized by increased plasma corticosterone levels, is reported to be associated with altered gut redox status and immune system [35,36]. We observed that CUMS-induced oxidative stress was concomitant to a decrease in enzymatic and non-enzymatic antioxidant levels. During oxidative stress, excessive ROS, by inducing protein oxidation, lipid peroxidation, and DNA damage, affect cellular function [35]. Thus, oxidative modification of the cellular proteins, indicated by high intestinal PCO content, might have inhibited the enzymatic antioxidants such as SOD and CAT in the intestinal mucosa (Figure 3). GSH is a powerful endogenous non-enzymatic antioxidant that is affected by intracellular ROS accumulation. Since GSH is an important endogenous antioxidant, a decrease in GSH level indicates oxidative assault. Cell membranes are protected by the GSH redox cycle from lipid peroxidation (LPO), a chain reaction in which polyunsaturated fatty acids (PUFA) present in membrane lipids are oxidized by ROS [37,38]. Therefore, the decreased GSH content may be responsible for the increase in the levels of MDA, a lipid peroxidation product, in the homogenate of intestinal mucosa of rats exposed to CUMS. Since oxidative stress plays a pivotal role in stress-induced pathogenesis, the suppression of oxidative stress, by the activation of antioxidant defense, could be an important mechanism to maintain intestinal integrity. A key function of the Nrf-2 pathway is to induce the intrinsic antioxidant response within cells to prevent redox imbalances. Inside the nucleus, Nrf-2 binds with antioxidant response elements and activates its downstream target genes, including HO-1 and NQO-1, which upregulate the synthesis of enzymatic and non-enzymatic endogenous antioxidants to counter cellular oxidative stress. Previous studies have shown that the CUMS-induced pathophysiology can be effectively reversed by probiotics supplementation [39]. Supplementation of *C. butyricum* and *B. infantis* to the rats exposed to CUMS significantly decreased the cortisol levels in the blood and showed enhanced protein expression of Nrf-2 and its downregulated target genes HO-1 and NQO-1, which may have subsequently resulted in the increased enzymatic and non-enzymatic antioxidant activities in the rat intestine. Further, the increased antioxidant defense may have protected the oxidant-induced protein and lipid damage to the intestinal tissue.

Increased oxidative stress triggers inflammation, which, in turn, magnifies the oxidative stress, and generates a circle resulting in cell damage and promoting a pro-inflammatory state [40]. Consistent with the previous studies, our results showed that long-term exposure to CUMS not only induced redox imbalance, but also significantly increased inflammatory mediators such as TNF-α and IL-6 levels in tissue homogenate. Exposure to CUMS showed enhanced intestinal protein expression of NF-κB, a transcription factor that regulates a variety of biological processes such as inflammation and immune response. We observed that CUMS-induced activation of the NF-κB was associated with increased production of inflammatory cytokines [41]. Along with the tissue inflammation, an increase in acute phase inflammatory mediators, such as IL-6, TNF-α, and CRP in blood plasma, shows an inflammatory response in the circulation. Nrf-2 not only acts as an upstream regulator of oxidative stress, but also suppresses inflammation by stimulating the regulatory mechanism against NF-κB activation (He et al., 2020). Nrf-2 and NF-κB signaling pathways cooperate to regulate cellular redox status and inflammation in response to stress [42]. Nrf-2-induced HO-1 activation causes inhibition of NF-κB mediated pro-inflammatory activity [43]. Hence, the probiotic-induced activation of Nrf-2 mediated suppression of the NF-κB protein expression might have inhibited the inflammation in the intestine. This is consistent with the decreased levels of TNF-α and IL-6 levels in the intestinal tissues (Figure 3).

It has been shown that stress increases gut permeability [11]. The impact of acute stress on intestinal permeability has also been confirmed in human studies [44]. Tight junctions have many complex structural and functional proteins, including occludin and claudin-1, which, along with ZO-1, regulate the function of intestinal permeability. It has been reported that exposure to stress can break down the intestinal mucosal barrier and enhance intestinal epithelial permeability due to changes in the tight junction proteins, which can initiate or exacerbate the onset of colon inflammation in a healthy individual [11]. CUMS-induced morphological changes showed loss of epithelial surface with loose and denuded villi (Figure 8), which is consistent with the results of a previous study that reported CUMS-induced histopathological alteration of colon tissue (Ding et al., 2020). Stress-induced alteration in the mucosal layer and damage of intestinal epithelium causes impairment of the intestinal barrier [45,46]. Lipids are major components of the plasma membrane. Oxidative stress-induced protein and lipid oxidation may have altered the structure and function of the tight junction protein, as well as damaged the intestinal epithelium, which compromises the integrity of the intestinal barrier [47]. Stress-induced disruption of the intestinal barrier can cause a leaky gut, and, subsequently, bacterial translocation and release of its endotoxins into the circulation, which could be the cause of systemic inflammation [48]. Studies have shown that the Nrf-2 pathway, by activating HO-1, can upregulate the protein expression of tight junction proteins occludin and ZO-1 [49,50]. Hence, activation of Nrf-2/HO-1 signaling and decreased NF-κB expression induced by probiotics, including *C. butyricum* and *B. infantis*, might have protected the intestinal mucosal injury and tight junction dysfunction by reducing the colonic inflammation and oxidative stress.

Immune tolerance is important to maintain intestinal homeostasis. Results of studies have revealed that stress-induced increased gut permeability can stimulate immune system alteration within the intestine [51]. A major population of immune cells in the intestine includes T-cells, mainly CD8+ and CD4+ T-cells [52]. We observed that CUMS modulated T-cells in rat’s intestine. This was demonstrated by a significant decrease in CD4+ T-cells with an increase of CD8+ T-cells in the CUMS exposed group (Figure 6 and Figure 7). This is in agreement with previous studies which reported that stress and altered humoral immunity are associated with decreased CD4+ T and increased CD8+ T-cells, promoting chronic inflammation by increased production of proinflammatory cytokines such as interferon-γ, TNF-α, and IL-6 [53,54]. Besides blocking inflammation, Nrf-2 regulates immune responses. In response to Nrf-2 activation, CD4+ T-cells shift toward Th2 differentiation, which stimulates IL-4, IL-5, and IL-13 production, which has an anti-inflammatory effect [55]. Thus, from our results, we speculate that a probiotic mixture of *C. butyricum* and *B. infantis*, by activating the Nrf-2 pathway, not only attenuated the CUMS-induced oxidative stress, but also inhibited NF-κB protein expression and Nrf-2-induced restoration of the immune modulation by increasing the level of CD4+ T lymphocytes, which might have further promoted the anti-inflammatory responses and protected mucosal injury and intestinal barrier integrity [55].

## 4. Material and Methods

The freeze-dried probiotic mixture of *C. butyricum* CGMCC 0313-1 (containing 2.4 × 10^8^ colony-forming units (CFU)/g viable bacteria) and *B. infantis* CGMCC 0313-2 strain (containing viable bacteria 1.8 × 10^9^ CFU/g), purchased from Shandong Kexing Bioproducts Co., Ltd., Jinan, Shandong, China, was used in the study. To check the viability, a randomly-chosen sachet of probiotics powder reconstituted in sterile saline was incubated anaerobically at 37 °C for 48 h on blood agar plates (Hardy Diagnostics, Santa Maria, CA). The result showed small white colonies growth in the plates. A probiotic mixture solution, including *C. butyricum* at concentration 1.0 × 10^8^ CFU/mL and *B. infantis* at concentration 1.0 × 10^9^ CFU/mL, was freshly prepared before the supplementation.

### 4.1. Animals and Experimental Design

Twenty-four male Wistar rats, weighing 200–250 g, used in the experiment were obtained from the animal house unit, Department of Zoology, Science College, King Saud University (KSU). Humane conditions were maintained for all animals as per international standards. The experimental protocol was approved by the Research Ethics Committee at the College of Applied Medical Sciences, KSU. Ten days before the experiment, the rats were stabilized on a standard pellet rat diet with free access to water. After the adaptation period, they were randomly divided into 4 groups of 6 rats per group.

Group I—Control animals were left undisturbed and handled gently except for daily weighing.
Group II—Probiotics group was orally supplemented with the probiotic mixture (4 × 10^7^ CFU *C. butyricum* and 4 × 10^8^ CFU B. infantis) suspension alone daily as a single dose for 28 days.
Group III—CUMS group rats were exposed to CUMS once daily for 28 days.
Group IV—CUMS + Probiotics group rats were, along with CUMS, orally supplemented with a probiotic mixture (4 × 10^7^ CFU *C. butyricum* and 4 × 10^8^ CFU B. infantis) suspension daily as a single dose for 28 days.

### 4.2. Stress Protocol

As previously described, rats were exposed to one of the following stressors daily for 28 days in an unpredictable manner, but with some modifications [56,57]. Exposure to the same stressor was not carried out for more than two consecutive days.

CUMS exposure: Tilted cage—home cages were tilted at a 45° angle for 5 h; Shaking—4 groups of rats were placed in a plastic box container and placed in an orbital shaker for 30 min at 150 rpm; Restraint—rats were placed in a 50 mL plastic tube (Falcon) with openings on both sides for breathing for 3 h; Social defeat—rats were placed in a transparent and perforated plastic container to avoid further physical contact for 8 h; Inverted light cycle—regular room light was switched off during the day time and put on during night time for 2 days.

The initial and final body weight of the rats were recorded during the experimental period.

### 4.3. Sample Collection and Processing

After 28 days of the experimental period, the rats were sacrificed to collect the blood samples and intestinal tissues for analysis. The blood samples were centrifuged at 3500 rpm for 10 min, and the plasma from each group was collected in labeled Eppendorf tubes and stored at −80 °C. After removing the entire small intestine, the luminal contents were flushed with ice-cold physiological saline. For histological analysis, a small intestinal segment from each group was fixed in neutral buffered formalin (10%) solution. The remaining intestine was completely slit open to gently scrap the mucosa with a glass slide for the preparation of 10% (*w*/*v*) homogenates in ice-cold PBS. The homogenized mucosa was centrifuged at 14,000× *g* (4 °C) for 15 min, and the supernatant thus collected was stored at −80 °C for further analysis.

### 4.4. Estimation of Corticosterone in Plasma

The concentration of plasma corticosterone was detected by the ELISA kit (Abcam, Cambridge, UK), and the concentration was expressed as ng of corticosterone per ml of plasma.

### 4.5. Estimation of Inflammatory Markers

The concentrations of C-reactive protein (CRP), as well as interleukin-6 (IL-6) and tumor necrosis factor-α (TNF-α) in the plasma, were detected by ELISA kits procured from Abcam, Cambridge, UK. The quantitative measurement of IL-6 concentration in the small intestine mucosal homogenate was estimated by the TNF-α and IL-6 Rat ELISA kit (Abcam, Cambridge, UK).

### 4.6. Detection of Oxidative Stress Markers

The concentration of enzymatic antioxidant catalase was estimated by the specific activity assay kit (Abcam, Cambridge, UK). Superoxide dismutase (SOD) activity was measured using a colorimetric SOD assay kit (Abcam, Cambridge, UK). Reduced glutathione (GSH), the non-enzymatic oxidative stress marker, was detected in the intestinal homogenate by glutathione fluorometric assay kit (Abcam, Cambridge, UK).

The level of lipid peroxidation was measured by malondialdehyde (MDA) level in intestinal homogenate using an MDA colorimetric/fluorometric assay kit procured from Abcam, Cambridge, UK. The intestinal protein carbonyl (PCO) content was estimated spectrophotometrically, in which 2,4-dinitrophenylhydrazine reacted with carbonyl groups to form 2,4-dinitrophenylhydrazone, and the results were expressed as nmol/mg protein [58].

### 4.7. Detection of Mucosal Immune Cells by Immunohistochemistry

The expressions of CD4+ and CD8+ T-cells in the intestinal mucosa were detected by immunohistochemistry. Sections of 5 μm thickness from paraffin-embedded rat intestine tissue blocks were prepared. The slides were initially deparaffinized, and, to each section, Peroxidase-Blocking Reagent (Agilent, Santa Clara, CA, USA) was added to block the endogenous peroxidase (10 min). The sections were incubated for 2 h with diluted primary Anti-CD4 antibody (1:100; ab33775; Abcam, Cambridge, UK) and Anti-CD8 antibody (1:100; ab203035; Abcam, Cambridge, UK). After incubation, the slides were washed with Tris-buffer-saline buffer and incubated with a secondary antibody for 30 min. Next, freshly prepared 3,3′-diaminobenzidine (DAB) was added (15 min). Slides were additionally stained with hematoxylin counterstain for 15 min, rinsed, and dehydrated in 75% and 100% absolute alcohol, and xylene. After gently placing a coverslip over the slides, the sections were examined microscopically for the presence of CD4+ and CD8+ T-lymphocytes. Further, quantitative analysis was performed on each CD4+T and CD8+T-lymphocyte stained section by counting positive cells manually in 20 random fields at a magnification of 400× on a computer monitor connected to an optical microscope (Olympus Optical BX51TF, Olympus, Tokyo, Japan).

### 4.8. Histopathological Evaluations

Small intestinal segments from each group were fixed in neutral buffered formalin solution (10%) and embedded in paraffin wax. Approximately 4–5 µm sections of the paraffin blocks were sliced and stained with hematoxylin and eosin. After, a cover slip was gently placed over the stained sections. Finally, all slides were analyzed using an optical microscope (Eclipse 50i; Nikon, Minato, Tokyo, Japan).

### 4.9. Western Blotting

A radioimmunoprecipitation assay (RIPA) lysis buffer (Boston Bioproducts, Ashland, MA, USA) containing phosphatase and protease inhibitors was used for protein extraction. The concentration of protein was estimated by Bradford assay. Subsequently, by using sodium dodecyl sulfate-polyacrylamide gel electrophoresis (12% SDS-PAGE), 25 μg of protein samples were separated. On nitrocellulose membranes, separated proteins were transferred, followed by blocking with 5% skim milk for 2 h at room temperature. Next, the blot was incubated overnight at 4 °C with the primary antibodies against occludin (1:500; sc-133256; Santa Cruz Biotechnology, Inc., Dallas, Texas, USA), claudin-1 (1:1000; sc-166338; Santa Cruz Biotechnology, Inc., Texas, USA), Nrf-2 (1:1000; ab92946; Abcam, Cambridge, UK), HO-1(1:1000; ab90515; Abcam, Cambridge, UK), NQO-1 (1:1000; sc-47778; Santa Cruz Biotechnology, Inc., Texas, USA), NF-κB (1:1000; ab32536; Abcam, Cambridge, UK), and β-actin (1:1000; sc-47778; Santa Cruz Biotechnology, Inc., Texas, USA). Following washing, secondary antibodies conjugated with horseradish peroxidase (HRP) were incubated for one hour. To detect antigen–antibody complexes, a pierce-enhanced chemiluminescence detection system (Thermo Fisher Scientific Inc., Waltham, Massachusetts, USA) was used, and the images were analyzed through ImageLab software 6.1 (Bio-Rad, Hercules, California, USA).

### 4.10. Statistical Analysis

All data are presented as mean ±SEM, using GraphPad Prism 9 software (GraphPad Software, Inc., San Diego, CA, USA) for statistical analysis. One-way analysis of variance (ANOVA) was performed to determine the significance among the groups, followed by Tukey’s post-hoc test. The *p* < 0.05 was considered statistically significant.

## 5. Conclusions

Exposure to CUMS leads to oxidative stress, increased inflammation, and T-lymphocyte alteration in the intestine. However, a probiotic mixture of *C. butyricum* and *B. infantis*, by activating Nrf-2/HO-1 signaling and repressing NF-κB protein expression, attenuated CUMS-induced oxidative stress and inflammation, altered T-lymphocytes cells, and preserved the intestinal epithelial integrity. Our findings highlight the beneficial effects of a probiotic mixture of *C. butyricum* and *B. infantis* as a functional food, and provide a new perspective on the prevention and promotion of intestinal health during exposure to chronic stress. Thus, consumption of a probiotics mixture, including *C. butyricum* and *B. infantis*, can be beneficial in various stressful situations in daily life to protect intestinal function and integrity.

## Data Availability

The corresponding author will provide data from the current study upon reasonable request.
